# Cadmium and Copper Stress Responses in Soapbark Tree (*Quillaja saponaria*): Effects on Growth, Metal Accumulation, Saponin Concentration, and Gene Expression

**DOI:** 10.3390/plants14050709

**Published:** 2025-02-26

**Authors:** Javiera Lazo, Jaime Tapia, Fernando P. Guerra

**Affiliations:** 1 Escuela de Bioquímica, Universidad de Talca, Talca 3460000, Chile; 2 Instituto de Química de Recursos Naturales, Universidad de Talca, Talca 3460000, Chile; 3 Instituto de Ciencias Biológicas, Universidad de Talca, Talca 3460000, Chile

**Keywords:** heavy metals, phytoremediation, quillay, saponins, stress-induced genes, tree species

## Abstract

Heavy metals such as Cu and Cd are important pollutants. Quillay (*Quillaja saponaria*) is a tree species endemic to Chile that is of worldwide commercial interest due to its saponins. It can grow on contaminated sites. However, the biological mechanisms underlying its defensive responses remain elusive. This study aimed to characterize Quillay plants under Cu and Cd stress and identify mechanisms controlling their interaction with these metals. We subjected six-month-old plants to Cu (75, 150, and 300 μM) and Cd (20, 40, and 80 μM) in hydroponics for a week and assessed growth, metal accumulation, saponin production, and the expression of a suite of stress-induced genes. Those genes are related to phytochelatins (*PCS*) and metallothioneins (*MT*), the antioxidant system (*GS* and *GR*), and metal transporters (*COPT1*). The results indicated that both metals were accumulated mainly in roots, with 339.9 and 433.8 mg/kg DW, for Cd and Cu, respectively, exhibiting a metal excluder pattern. Cd increased the length of the principal root. Higher doses of Cd and Cu augmented the saponin content (62.8% and 41.2% compared to control, respectively). The genes *GS*, *GR*, and *COPT1* modified their transcriptional levels depending on the metal and organ evaluated. These results provide evidence of specific defensive responses of this species against heavy metal stress, which is helpful to guide new research efforts and support the development of strategies for using Quillay for phytoremediation.

## 1. Introduction

Heavy metals, such as cadmium (Cd) and copper (Cu), are naturally present in soils but can become significant pollutants due to human activities such as mining, smelting, combustion of fossil fuels, phosphate fertilization, and the addition of sludge to soils, among others [1,2]. Contaminated agricultural lands (and water resources) are particularly concerning, as they impact ecosystems, crop yield, and food security. Due to their bioaccumulation ability, these metals pose serious health risks to humans and animals. This situation is critical in mining countries, like Chile, where Cu and Cd represent environmental and public health challenges, affecting marine and terrestrial ecosystems and human communities [3,4,5]. In the case of plants, some heavy metals, such as Cu or Fe, are essential nutrients. Others, such as Cd and Pb, are not required for metabolism but can be updated and accumulated in plant organs. With different toxicity profiles, high concentrations of both types of metals can produce adverse effects. An excess of these elements can severely impair their growth, leading to symptoms such as chlorosis, necrosis, and inhibited root development [6,7].

Phytoremediation utilizes plants and their associated microbes for environmental cleanup [8]. This technology is suitable for remediating soils contaminated with heavy metals cost-effectively and environmentally friendly [9]. Native and introduced invasive plant species have been proposed as resources for this purpose [10,11]. One of the more critical characteristics that plants must meet is tolerating the metal concentrations they accumulate [12]. Plants have evolved homeostatic mechanisms to deal with variations in the level of essential and non-essential elements and reduce the damage associated with oxidative stress and the osmotic imbalance induced by excess elements [13,14]. These mechanisms operate from the cells to the whole plant. Protein transporters play an essential role in regulating the uptake and movement of metals across membranes and the compartmentation into cell organelles or plant organs [15,16]. A characterization of different transporter proteins was reported recently elsewhere by Huang et al. [17]. Some of these transporters have been associated with the movement of Cu as the Copper Transporter (COPT) and Iron-Regulated Transporter-1 (IRT1) [15,16]. Plant cells have no specific Cd transporters; however, this metal moves, taking advantage of Fe, Zn, and Mn membrane transporters [17]. In addition, the cell defense system, comprising enzymes related to the biosynthesis of antioxidant metabolites, such as glutathione, glutathione synthetase (GS), and glutathione reductase (GR), among others, plays a crucial role in mitigating the oxidative stress caused by reactive oxygen species (ROS) that accumulate during metal exposure [18]. Furthermore, metal-chelating molecules like phytochelatins (PCs) and metallothioneins (MTs) bind to and neutralize heavy metals in the cytoplasm, protecting plant cells against their chemical reactivity and potential ROS production [19]. This function is based on multiple cysteine residues in their respective structures. Phytochelatins are synthesized from glutathione by the enzyme phytochelatin synthase (PCS), whereas MTs are the product of the expression of their encoding genes [20,21]. The main findings in this research area come from studies based on herbaceous model species, including *Arabidopsis thaliana*, *Oryza sativa*, *Hordeum vulgare*, and *Thlaspi caerulescens*, among others [15,16]. Forest tree species have been less intensively evaluated regarding their mechanisms and suitability for phytoremediation. Higher biomass production (including wood), long-term life cycles, clonal propagation for some species, and their deep rooting, favoring the metal extraction from deeper soil layers, make these plants attractive for optimizing phytoremediation systems on heavy metal-contaminated sites [22,23]. Different biological and operational aspects of using fast-growing trees for phytoremediation of heavy metals have been reviewed by Pajević et al. [24] elsewhere. In that context, understanding the adaptive responses of tree species subjected to heavy metal-induced stress and evaluating candidate species are critical for developing and improving phytoremediation plantations.

The soapbark tree, or Quillay, *Quillaja saponaria* Mol., is an evergreen species belonging to the Quillajaceae family (Fabales order) that is endemic to the Mediterranean regions of Chile, forming part of sclerophyllous forests [22,25]. It is considered a species adapted to dry and warm climates and can persist in wet and snowy places [26], forming mixed forests [27]. This species can also tolerate poor soil and drought conditions [25]. Quillay has been traditionally recognized for its high production of saponins, which are mainly extracted from its bark. These metabolites have a high commercial interest in pharmaceutical (including vaccine production against SARS-CoV-2), food, animal production, and cosmetic areas [22,28,29,30]. In recent studies, Quillay has been studied in Chile in terms of its growth on Cu mine tailings and surrounding sites contaminated by heavy metals, and it has been recommended as a candidate species for phytostabilization [31,32]. The ability of Quillay to grow successfully under those conditions suggests the influence of some tolerance mechanisms. However, the knowledge about them is minimal.

Saponins are high molecular weight glycosides produced by the plant’s secondary metabolism. These metabolites promote plant immunity against insects, pathogens, and herbivores and could be involved in adaptive changes to survive in adverse environmental conditions [22]. Regarding the interactions with metals, saponins can bind heavy metals by their carboxyl and hydroxyl functional groups, generating ion exchange and complex formation [33]. The metal adsorption or chelation by isolated saponins from plants, such as the tea plant (*Camellia sinensis*), has been applied to enhance the removal of heavy metals from contaminated soils [34,35,36]. Isolated Quillay saponins have been used in hydrometallurgical processes to recover copper [37]. As Quillay has not specifically evolved in metalliferous soils, its tolerance to heavy metals is likely driven by its ability to adapt to abiotic stress. Therefore, we hypothesize that Quillay copes with heavy metal stress through saponin production metal homeostasis and oxidative stress protection mechanisms. Within this framework, and considering the environmental importance of Cu and Cd in Chile, our objectives were to assess the effects of Cu and Cd on the growth, metal accumulation patterns, saponin production, and gene expression of Quillay and identify mechanisms controlling its interaction with these heavy metals. Plants (seedlings) were established in a hydroponic assay under controlled conditions (in a growth chamber). Different traits and gene transcript profiles were evaluated after one week of exposure to different metal doses. We employed an approach combining various traits and targeted gene expression analyses. To our knowledge, this integrative methodology has not yet been applied to this species. Our findings suggest that saponins play a defensive role against heavy metal stress in plants, a novel aspect of plant research. Additionally, we gathered valuable information on saponins and metal accumulation, which is relevant for industries requiring high-purity saponins.

## 2. Results

### 2.1. Metals Modified Root Growth and Induced an Excluder Accumulation Pattern

**Figure 1:**
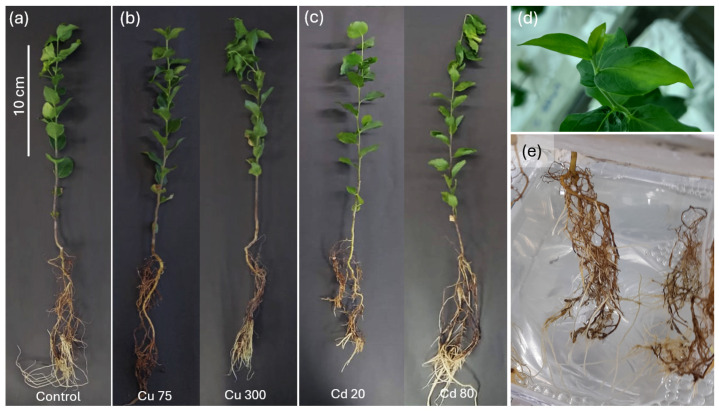
The general growth of representative Quillay plants exposed to metal stress for one week. (**a**) Control solution. (**b**) Copper stress (75 and 300 µM). (**c**) Cadmium stress (20 and 80 µM). (**d**) Detail for leaves of plants exposed to Cu 300 µM exhibiting chlorosis. (**e**) Detail for roots of plants exposed to Cd 20 µM. Plants corresponded to six-month seedlings (they were grown for 5 months in pots with organic substrate and one month on hydroponics). For the treatments represented in (**b**,**c**), no significant differences were detected in plant height compared to the control.

Figure 1 illustrates the general response of soapbark plants subjected to stress from Cu and Cd. Qualitative differences compared to the control plants were observed at higher doses of both metals. Cu at a dose of 300 μM produced root browning, partial leaf chlorosis, and slight stem wilting (Figure 1b,d). The Cd concentration of 80 μM had similar effects on aerial organs as the Cu dose of 300 μM. However, root growth and leaf color were unaffected (Figure 1c,e).

**Figure 2:**
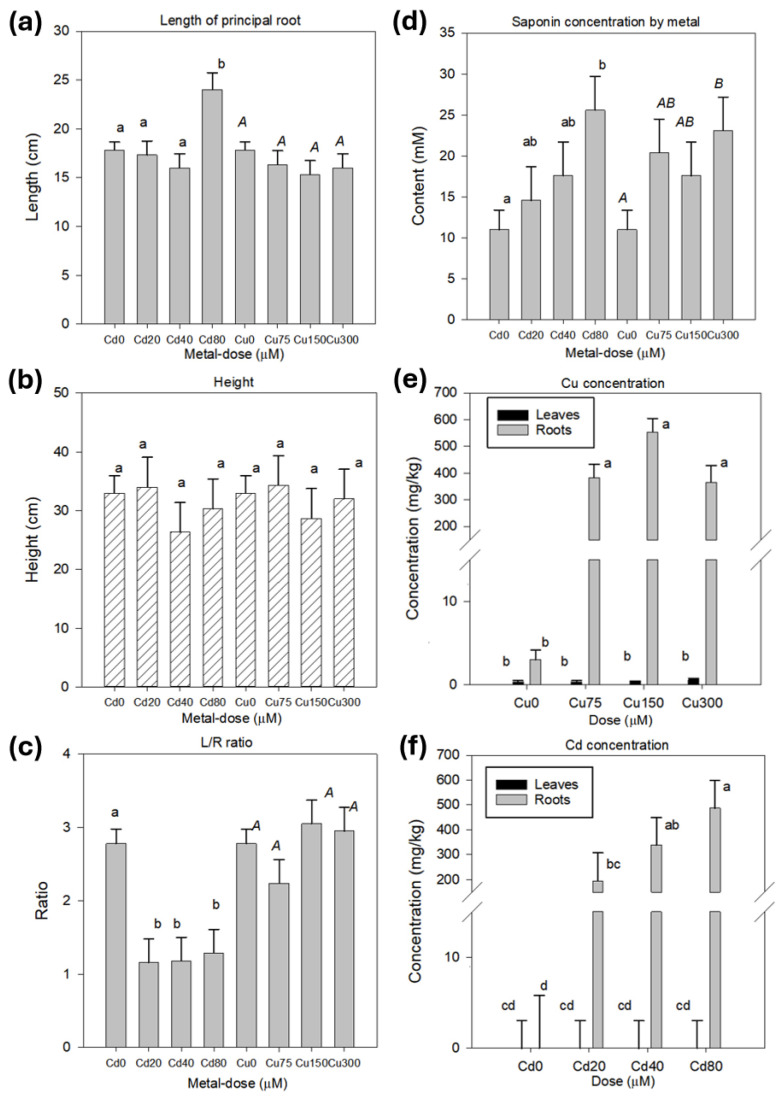
Growth traits, saponin, and metal concentrations in Quillay plants exposed to metal stress. (**a**) Length of principal root (LPR). (**b**) Plant height. (**c**) Leaf-to-root (L/R) biomass ratio. (**d**) Saponin concentration by metal and dose within the metal. (**e**) Cu concentration by organ and Cu treatments. (**f**) Cd concentration by organ and Cd treatments. Bars represent least square means with standard errors. Different letters indicate significant differences (least-squares mean differences Tukey HSD, *p* < 0.05) among metal doses or organs.

The analysis of variance (ANOVA) for growth and concentration traits revealed significant effects (*p* < 0.05) of the factors metal, dose within metal, and plant organ, depending on the specific variable analyzed (Table 1). Table 2 and Table 3 show the observed averages for these three factors. Metal exposure significantly influenced root morphology, particularly the length of the principal root (LPR) and the leaf-to-root (L/R) ratio (Table 1 and Table 2; Figure 2a,c). Specifically, both traits were modified by Cd exposure (but not by Cu), producing an increase (nearly 5 cm) in LPR at the 80 μM dose, equivalent to a 34.6% increase compared to the control and a decrease (nearly 43%) in the L/R ratio for all the evaluated doses (Table 2; Figure 2a,c). On the other hand, the plant height and weight (fresh weight [FW] and dry weight [DW]) were not altered by the metals (Table 1 and Table 2). In the case of both FW and DW, significant differences were observed only between roots and leaves.

The results showed that the concentration of Cu and Cd in Quillay plants depended on the dose of each metal and organ (Table 1). Exposure to Cu and Cd produced a significant accumulation of these metals which was higher in roots than in leaves (Table 3). In roots, Cu concentrations increased from 3.02 mg/kg (control) up to 433.8 mg/kg (average for all the assessed doses) (Table 3, Figure 2e). Cd was not detected in the roots of the control plants. However, applying this metal in different amounts significantly increased its concentration to 339.9 mg/kg (average for all the assessed doses) (Table 3). That rise was proportional to the applied dose (Figure 2f). Regarding leaves, no significant differences were observed between the control and metal (Cu and Cd)-exposed plants. Significant amounts of Cd were not detected in the leaves of the plants evaluated (Table 3). The predominant accumulation of metals in the roots indicates a pattern of exclusion from the aerial structures.

### 2.2. Metal Stress Increased the Saponin Content

The saponin content showed significant effects on plant organs at different doses within each metal (Table 1). Table 3 indicates that the concentration of these metabolites was nearly four-fold higher in leaves than in roots. Furthermore, on average, exposure to Cu and Cd resulted in saponin levels that were approximately two-fold higher than those in the control plants. Figure 2d illustrates an increase in saponin concentration corresponding to the doses of each element. Specifically, the highest doses of Cd (80 µM) and Cu (300 µM) led to a significant rise in saponin content. These contents reached 25.6 mM for Cd and 23.1 mM for Cu, representing increases of 62.8 and 41.2% compared to the control for each metal, respectively.

### 2.3. Saponin Content Was Associated with Metal Concentration

Table 4 presents the results of the correlation analysis for the studied traits, revealing significant relationships between some of them. Plant height was significantly associated with leaf and root weights (FW and DW) and with the L/R ratio, with correlation values ranging from r = 0.44 (L/R ratio) to r = 0.92 (leaf weight). Leaf and root weights (fresh and dry) were positively and significantly correlated. A significant correlation (r = 0.59) was observed regarding the metal concentration in the organs between the root and leaf Cu contents. No significant correlations were observed for metals and growth traits, except for the L/R ratio, root DW, and Cd in roots. A significant negative correlation (r = −0.70) was detected for the L/R ratio and Cd, and a positive relationship between root DW and Cd (r = 0.47). These last correlations are consistent with the higher root growth observed under Cd application (Figure 1e and Figure 2a).

Interestingly, the saponin content varied with metal concentrations in different plant organs. In particular, the root concentrations of Cu and Cd were positively correlated with saponins in leaves, with r = 0.56 and r = 0.62, respectively. In addition, the root concentration of Cu was negatively correlated with the saponin concentration in the same organ (r = −0.47). The saponin concentration was not correlated with any of the growth traits analyzed.

### 2.4. Analysis of Gene Expression Indicated an Organ-Specific Regulation

**Figure 3:**
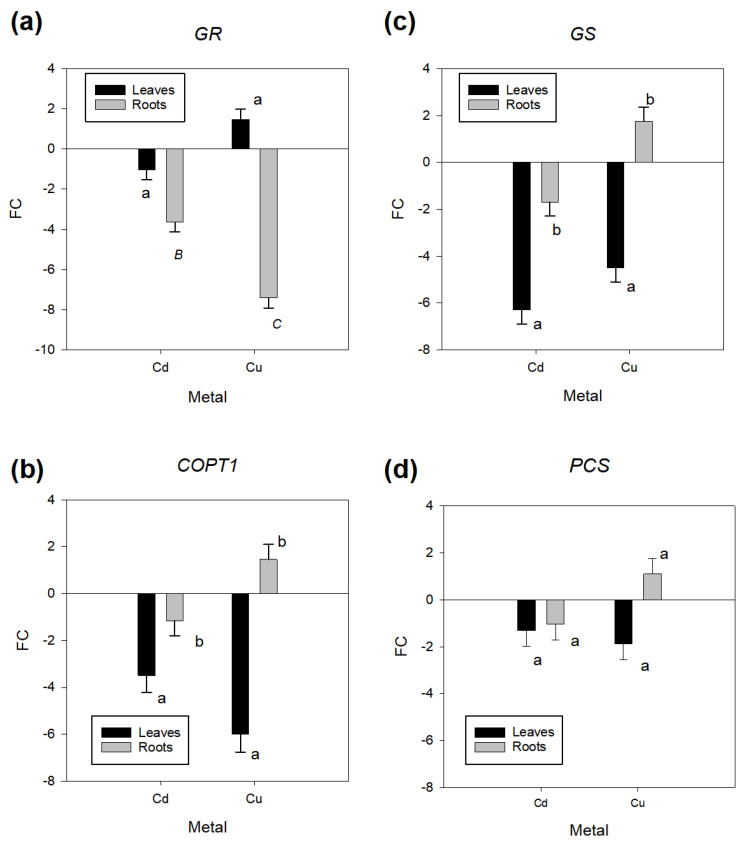
Transcriptional variation of four genes in Quillay plants exposed to Cu and Cd. (**a**) Glutathione reductase (*GR*). (**b**) Glutathione synthetase (*GS*). (**c**) Copper transporter (*COPT1*) and (**d**) Phytochelatin synthase (*PCS*). FC represents the fold of change regarding control plants. Different letters indicate significant differences (least-squares mean differences Tukey HSD, *p* < 0.05) among metal levels or organs.

At the transcript level, we analyzed the expression of genes encoding enzymes involved in the antioxidant system, membrane transport, and metal chelation. Table 5 shows that the genes encoding the glutathione metabolism enzymes *GR* and *GS* exhibited significant transcript differences between plant organs. Under Cu exposure, the *GR* gene was upregulated in leaves (1.5-fold change [FC]) but significantly downregulated in roots (−7.5 FC) (Figure 3a). Cadmium stress downregulated this gene in roots (−3.7 FC) and leaves (−1 FC) (Figure 3a). When comparing metals, the downregulation of *GR* in roots was more pronounced under Cu than under Cd. Regarding *GS*, this gene was upregulated in roots (1.8 FC) but downregulated in leaves (−4.5 FC) in plants subjected to Cu stress. Under Cd exposure, this gene was downregulated in both organs, with −6 and −1.5 FC in leaves and roots, respectively.

Regarding genes encoding metal transporters, Table 5 indicates that *COPT1* exhibited differential regulation between plant organs. Under Cu exposure, this gene was upregulated in roots (1.7 FC) and downregulated in leaves (−6 FC) (Figure 3b). In contrast, Cd produced a downregulation in both organs (−3.5 FC in leaves and −1 FC in roots) (Figure 3b). The expression of *COPT1* was significantly affected by different doses of Cu, particularly in roots (no differences were observed among doses in leaves, with an average of −6 FC). Specifically, Cu 75 μM significantly downregulated this gene −7.2-fold, whereas Cu 150 μM and 300 μM upregulated *COPT1* by 3.1-fold and 73-fold, respectively (Figure S1). For genes involved in producing metal chelating molecules, in the case of PCS, no significant effects on *PCS* transcript levels were observed in exposed plants (Table 5, Figure 3d). *MT* transcripts were detected only in leaves, where both Cd and Cu exposure led to gene downregulation (−5.5 and −360 FC, respectively) (Figure S2).

The correlation analyses among transcript profiles for the different genes showed a positive and significant (*p* < 0.05) association among *COPT1*, *GS*, and *PCS* under both metals, indicating a similar expression pattern (Figure S3). For Cu, *COPT1* correlated with *GS* (r = 0.74) and *PCS* (r = 0.69). *GS* was also significantly associated with *PCS* (r = 0.51). Under Cd stress, *GS* was correlated with *COPT1* (r = 0.71) and *PCS* (r = 0.67). Transcription of the *GR* gene was not significantly associated with any other gene under Cu or Cd stress.

## 3. Discussion

### 3.1. Growth Responses to Heavy Metal Stress

Despite the well-documented phytotoxicity of Cd and Cu in many plant species [38,39,40], Quillay plants showed limited visible symptoms of stress. Both aerial and root dry weights did not significantly differ between the metal-treated and control plants, suggesting a baseline tolerance to these heavy metals. However, some qualitative changes were observed in plants exposed to 300 μM of Cu (Figure 1b), including root browning, slight leaf chlorosis, and wilting. These effects are consistent with the known impact of excess Cu on root health and chlorophyll disruption in leaves [6,16]. These results suggest that Cu at 300 μM could represent the stress threshold for Quillay under the evaluated conditions. Interestingly, Cd produced a root elongation (LPR), with a significant difference at 80 μM and a consequent increase in the leaf-to-root ratio (Table 1, Figure 1 and Figure 2). An opposite response has been reported in other studies with tree species (belonging to the *Populus* and *Salix* genus), in which the LPR was significantly affected by Cd treatment (50 μM) [41]. The stimulated root growth in Quillay plants indicates a hormetic response, where low to moderate stress enhances growth in some plant species [39]. This adaptive reaction, driven by ROS accumulation, may be modulated by calcium movement and auxin-mediated pathways [42].

### 3.2. Heavy Metal Accumulation

The metal concentration results showed that Cu and Cd were predominantly accumulated in roots, with minimal translocation to aerial tissues (Table 2, Figure 2), reflecting a root-based exclusion strategy [19]. This pattern aligns with findings from a study on 6-year-old Quillay plants grown in a trial conducted on Cu mine tailings in Chile [32] and several tree species belonging to the *Populus* genus [43,44,45]. The restricted location of metals in roots is tree species’ most common resistance trait [23]. The exclusion mechanism likely involves apoplastic binding and vacuolar sequestration in root cells, which limits the upward transport of metals and reduces potential damage to photosynthetic tissues [46,47]. Particularly for Cd, the lack of translocation to leaves may also result from specific competition with calcium uptake during xylem loading [39]. Our study used calcium to precipitate sulfate from the Cu and Cd salts to prepare the stress solutions. Concerning specific organs, Cd was not detected in leaves in our study. The same result was observed by Milla-Moreno and Guy [32] for Quillay plants assayed on copper mine tailings. A total of 0.3 mg/kg of this metal was detected in Quillay leaves of plants that naturally colonized copper mine tailings in Central Chile [31]. Additionally, for the same organ, the Cu concentrations in our study (0.43 mg/kg average for treated plants) were lower than those reported by Milla-Moreno and Guy [32] (7.7 mg/kg) and Espinoza et al. [31] (18.5 mg/kg) for Quillay grown on mine tailings. Regarding roots, the average amounts for treated plants (339.90 and 433.79 mg/kg for Cd and Cu, respectively) were higher than those reported by Milla-Moreno and Guy [32] (0.18 and 264.1 mg/kg for Cd and Cu, respectively) for Quillay plants established on mine tailings. The lower root concentration for Cd in that study is noteworthy; however, these authors did not describe the referential content of this metal in the tailings. Different Quillay genotypes, ages of plants, and experimental conditions could be part of the factors explaining the differences among studies. Concerning other tree species and comparing with pure and hybrid species from the *Populus* and *Salix* genus exposed to 50 μM Cd in hydroponics, our concentration in leaves and roots was lower than that reported by Zacchini et al. [41] (ranging from 4296 to 9962 for roots, and from 293 to 651 mg/kg DW for aerial parts). A similar trend was observed when our results were compared with the Cd concentrations reported for other *Populus* species [48]. Regarding Cu content, for different *Populus* hybrids exposed to Cu concentrations of 8 and 16 μM under similar experimental conditions to our study, Cornejo et al. [43] determined average concentrations ranging from 335.8 to 1437.7 mg/kg in roots and from 40 to 45 mg/kg in leaves, which are higher than levels observed in our plants. Differences would suggest genetic variation among species and the impact of specific treatments.

### 3.3. Saponin Production

Saponin concentration was augmented in both leaves and roots in response to increased metal exposure (Table 1, Figure 2), suggesting that these metabolites might play a role in their adaptive response and, eventually, their metal tolerance. The significant correlation observed between root concentrations of Cu and Cd and saponin content in leaves (Table 4) suggests that the accumulation of these metals in roots could be a process priming the defensive role of saponins in leaves (sensitive organs to metal excess and ROS accumulation). However, further studies quantifying, for instance, molecules associated with oxidative stress effects, such as malondialdehyde or hydrogen peroxide, and evaluating their correlation with saponin contents and growth parameters will contribute to testing this hypothesis. Furthermore, the ability of saponins to chelate metals within the cells or tissues, reducing their presence as free ions, like MTs or PCs, deserves further analyses, considering their chemical structure, metal-binding properties, and diversity. The unchanged transcription rate of the *PCS* gene and the limited detection of *MT* gene transcripts suggest that these metal-chelating molecules were not actively involved under the tested conditions. This evidence supports the possibility that a different molecule may fulfill this function. In that sense, future studies (e.g., based on structural modeling) will be required to confirm the role of saponins in metal chelation. Foam formation was also observed in the growth media under Cd treatments, suggesting possible saponin exudation by the roots. Additional studies are needed to confirm their role in metal chelation within the rhizosphere. Also, some saponins are known to form stable complexes with heavy metals through their polar functional groups, making them potential agents for complementing the management of soils (or substrates) in phytostabilization and phytoextraction systems [34,35]. Furthermore, because quillay saponins would chelate heavy metals, it is important to verify through field trials if saponins of commercial interest (e.g., the QS-21 fraction [29]) could carry heavy metals when plants of this species are cultivated on contaminated soils.

### 3.4. Regulation of Heavy Metal Stress-Induced Genes

Gene expression analyses revealed the downregulation of *GS* and *GR*, regulatory components of the antioxidant system, in both roots and leaves under metal stress. This regulation suggests a potential imbalance in the glutathione–ascorbate cycle, which could reduce the capacity to neutralize ROS and maintain redox homeostasis [39]. The downregulation of these genes, especially under Cd stress, is consistent with previous reports on the depletion of glutathione pools during prolonged metal exposure [6]. Compared to other tree species, Ding et al. [49], analyzing the response of *Populus* × *canescens* grown in pots and exposed to Cd (75 μM), observed an upregulation and no changes for the *GS* gene in roots and leaves, respectively. This pattern of change, different from that observed in our study, reflects diverse dynamics at the level of both gene regulation and biosynthesis or depletion of glutathione, which depends on the plant species and specific experimental conditions. Further studies to analyze the effects of ROS (e.g., on lipid peroxidation), the activity of antioxidant enzymes, the accumulation of antioxidant metabolites (e.g., glutathione and ascorbate), and the interaction with sulfur [50], nitrogen [51], or sugar [52] metabolisms, can provide additional levels of information to confirm and complement our current findings. Interestingly, the *PCS* gene did not exhibit significant changes in expression, indicating that phytochelatin production may not be a primary defense mechanism in Quillay under the assessed conditions. The same pattern was observed in *Populus* × *canescens* plants exposed to Cd 75 μM [49]. No changes in PC biosynthesis could imply that other metabolites might have a role in metal chelation. In that sense, saponins might partially substitute PCs in directly interacting with metal ions and their detoxification in Quillay, a hypothesis that also deserves complementary studies. *COPT1*, responsible for Cu transport, was upregulated in roots but downregulated in leaves under Cu and Cd exposure. This pattern could involve a strategy to limit metal accumulation in leaves, protecting key processes such as photosynthesis [19]. Similar organ-specific gene regulation was observed by Zhang et al. [53] in tissue-cultured plantlets of *P. trichocarpa* subjected separately to Cu and Cd stress. These authors observed that the responses varied depending on specific members of the COPT gene family. The upregulation of *IRT1* in roots analyzed exploratorily in the assessed Quillay plants (Figure S4) further supports the role of specific transporters in controlling metal uptake and distribution within woody plants [47,54].

### 3.5. Future Prospects

Our results indicated that Quillay employs a defensive mechanism to exclude Cd and Cu from aerial organs under stress conditions. Additionally, the increased saponin levels and differential regulation of genes involved in the antioxidant system and membrane transport suggest further regulatory responses. Future studies will contribute to a broader characterization of these mechanisms. Those studies might include (1) quantification of the contents of antioxidant metabolites (e.g., glutathione and ascorbate), the activity of antioxidant enzymes (e.g., superoxide dismutase, catalase), or lipid peroxidation (by a malondialdehyde test); (2) an analysis of physiological parameters (photosynthesis and transpiration) and the interaction with sugar and nitrogen metabolisms; (3) an evaluation of the ability of Quillay saponins for chelating heavy metal ions (for instance, using bioinformatic modeling); (4) “omics” approaches (genomics, transcriptomics and metabolomics) focused on analyzing DNA polymorphisms and genetic diversity, whole gene expression profiles, and variability of primary and secondary metabolites; (5) the assessment of responses in plants representing other developmental stages, and different experimental environments (e.g., field trials on sites with heavy metal contamination). Considering the already-known use of saponins from other plant species for removing heavy metals from soils, it would also be interesting to evaluate the suitability of saponins isolated from Quillay for that purpose. The knowledge from these research lines will allow us to understand the biological basis of Quillay’s response to heavy metals and guide efforts for its genetic improvement for phytoremediation applications.

## 4. Materials and Methods

### 4.1. Plant Material, Growth Conditions, and Stress Treatments

Five-month-old Quillay seedlings, produced in plastic tree pot cells, were used for the experiment. The growth substrate (composted Monterrey pine bark) was carefully removed, and the roots were washed before placing the plants in a hydroponic system. That system consisted of an array of independent plastic units (boxes) containing 5 L of nutrient solution each. Six plants were placed within each unit. The nutrient solution corresponded to Hoagland’s modified basal salt mixture (PhytoTech Labs, Lenexa, KS, USA) at 0.25× strength, diluted in distilled water. After placing the plants in the system, they grew for one additional month before stress treatment. During this period, the nutrient solution was replaced every 3 days, and air pumps applied permanent oxygen flux to the solution. The experiment was conducted in a growth chamber at 18 °C, with a photoperiod of 12 and 8 h of light and darkness, respectively, and a photosynthetically active radiation of 57 mmol m^−2^ s^−1^.

Solutions of Cu and Cd were applied independently to the nutrient solution. Doses for Cu included 75, 150, and 300 μM. For Cd, the applied doses were 20, 40, and 80 μM. These stress solutions were prepared separately from CuSO_4_ or CdSO_4_ salts on one g L^−1^ Ca(NO_3_)_2_ solution. This set of doses was defined from preliminary hydroponic trials (under the same conditions as the experiment), and all of them were sublethal for both Cu and Cd. The entire solution was replaced every 3 days, and the plants were evaluated after one week under these conditions. This evaluation period was defined to avoid the excessive degradation of RNA in the exposed plants, which is proportional to their damage, and, in that way, to guarantee the gene expression analyses. Samples (leaves and roots) for the different measurements were collected and processed immediately or stored at −80 °C until analyzed.

The experiment was based on a factorial design (see details below), with metals (Cu or Cd) and doses within each metal as main factors, including three replicates and experimental units considering three seedlings each.

### 4.2. Phenotypic Measurements

The length of the stem (height) and the length of the principal root (LPR) were measured in each plant after the stress treatment and before being processed for different analyses. The LPR and similar parameters have been utilized previously as a primary indicator of metal tolerance [55,56]. Three plants per metal dose were used to measure fresh (FW) and dry (DW) weights. After drying, the same plants were analyzed to determine metal concentrations. The selected plants were chosen to represent each treatment in terms of their homogeneous size and general appearance. For DW, an estimator of biomass, plant leaves, and roots were separated and dried in an oven (Memmert ΜM300, Memmert, Schwabach, Germany) at 60 °C until a constant weight was achieved.

### 4.3. Metal Determinations

The concentration of Cu and Cd was determined independently in roots and leaves. One gram per sample was ground in a porcelain mortar, weighed, and subjected to acid digestion with HNO_3_ on hot plates under a hood until dryness was achieved, according to Tapia et al. [57]. Although a high-concentration mixed acid has been utilized to digest plant tissues [58], in our previous studies [57,59], concentrated HNO_3_ has produced more crystalline solutions for plant matrices. This situation has not been the case with sediments or animal tissues, where a mixture of acids is more effective than plant samples. The determinations were carried out by atomic absorption spectroscopy with air/acetylene flame (Unicam, Solaar 969, Thermo Fisher Scientific, Waltham, MA, USA). The detection limits for Cu and Cd were 0.0045 and 0.0028 mg L^−1^, respectively. The metal determination accuracy was validated using the certified reference material BIMEP 432 (Leyland cypress) from the Wageningen Evaluating Programs for Analytical Laboratories (WEPAL, Wageningen, The Netherlands). The quality controls using this reference material showed relative errors of 1.98% for Cu and 3.08% for Cd, respectively. The detailed results of the quantification validation, including the recovery rates, are presented in Table S1.

### 4.4. Saponin Quantification

The concentration of total saponins was determined in leaves and roots separately. The quantification for each experimental point was conducted using nine replicates (three biological × three technical). A total of 300 mg per organ sample was ground using a tissue homogenizer (IKA T25 Ultra-Turrax, IKA, Staufen, Germany) and water, and the mix was centrifuged for 10 min at 6000 revolutions per minute (Heraeus Megafuge 16R, Thermo Fisher Scientific, Waltham, MA, USA) to separate the solid and liquid phases. The supernatant was subjected to a reaction with 8% vanillin (Sigma-Aldrich CAS121-33-5, Milwaukee, WI, USA) and H_2_SO_4_ (72%), and the saponin content quantified by a UV–visible spectrophotometer (Jenway 6300, Jenway, Staffordshire, UK) at 545 nm, according to Hiai et al. [60]. A calibration curve was established using a purified *Quillaja* sp saponin extract as a standard (Sigma-Aldrich CAS 484 8047-15-2, Milwaukee, WI, USA) to relate absorbance to concentration. The concentrations of this extract used for modeling the curve ranged from 0.0250 to 0.1725 mM. The basic principle behind this quantification method is the reaction of oxidized triterpene saponins with sulfuric acid with vanillin, which gives a distinctive purplish-red color and can be measured at wavelengths ranging from 473 to 560 nm. Specifically, the sulfuric acid/vanillin reaction allows for the quantification of only the triterpene nucleus, corresponding to the OH at C-3 in free form, which is obtained by the hydrolysis reaction of the saponins with sulfuric acid and which subsequently reacts with vanillin [61].

### 4.5. Gene Expression Analysis

The gene expression analysis, at the transcript level, was performed by quantitative Real-Time PCR (qPCR) for the genes glutathione reductase (*GR*), glutathione synthetase (*GS*), metallothionein (*MT*), phytochelatin synthase (*PCS*), and copper transporter 1 (*COPT1*). According to the manufacturer’s protocol, total RNA isolation from leaves and roots was carried out using the Spectrum™ Plant Total RNA Kit (Sigma-Aldrich, Milwaukee, WI, USA). The isolated RNA was treated with DNase (Turbo DNA-free™ Kit Turbo™; Ambion, Austin, TX, USA), and the cDNA synthesis was carried out using the RevertAid First Strand system (Thermo Fisher Scientific, Waltham, MA, USA). The primers utilized for the qPCR reactions were based on gene sequences obtained from the NCBI (https://www.ncbi.nlm.nih.gov/, accessed on 15 December 2023) and Phytozome (https://phytozome-next.jgi.doe.gov/, accessed on 20 November 2023) platforms for taxonomically-related species. The design and efficiency of those primers were determined using Primer-Blast (https://www.ncbi.nlm.nih.gov/tools/primer-blast/, accessed on 20 November 2023) and Beacon Designer (http://www.premierbiosoft.com/qOligo/Oligo.jsp?PID=1, accessed on 20 November 2023), respectively. In addition to the target genes, two standardization genes, actin (*Act*) and glyceraldehyde-3-phosphate dehydrogenase (*GADPH*), were also used in the analysis. The primer sequences are included in the Supplementary Materials (Table S2). The qPCR reactions were carried out using the Brilliant II SYBR^®^ Green QPCR Master Mix kit (Agilent Technology, Santa Clara, CA, USA) in an AriaMx Real-time PCR System (Agilent Technology, Santa Clara, CA, USA). The thermal profile consisted of an initial denaturation at 95 °C for 3 min (one cycle), followed by 40 cycles of denaturation at 95 °C for 10 s, annealing at 60 °C for 30 s, and extension at 72 °C for 20 s. Finally, the melting curve included three steps at 95 °C for 30 s, 65 °C for 30 s, and 95 °C for 30 s. The variation in the relative abundance of transcripts was evaluated utilizing the 2^−∆∆Ct^ method [62,63]. For each target gene and experimental treatment, the ΔCt values were calculated as the difference between the target gene and the average of the standardization genes. The ΔΔCt values were estimated as the difference between the ΔCt determined under stress and control conditions. Three replicates per treatment were included.

### 4.6. Experimental Design and Statistical Analyses

According to the different evaluated traits, three linear models were applied, as described below. Nine replicates were used for each treatment group, with three allocated for phenotypic and metal determinations, three for saponin quantification, and three for gene expression analyses. The effects of the organ and metal doses on the Cu and Cd concentrations were assessed independently for each metal using the following model (associated with a two-way ANOVA):

Y_ijk_ = µ + O_i_ + D_j_ + OxD_ij_ + e_ijk_, (1)

where Y_ijk_ is the metal concentration (Cu or Cd) in the k-replicate and the i-organ under the application of the j-metal. µ is the general mean, O_i_ represents the fixed effect of the organ, D_j_ is the fixed effect of the applied metal dose, OxD_ij_ is the interaction between both factors, and e_ijk_ is the random effect of the residual.

The effects of metals and doses on plant height, LPR, and L/R ratio were considered in the following model (associated with a nested one-way ANOVA):

Y_ijk_ = µ + M_i_ + D(M)_j(i)_ + e_ijk_, (2)

where Y_ijk_ is the measurement in the k-replicate and the i-metal and j-dose. µ is the general mean, M_i_ represents the fixed effect of metal (Cu or Cd), D(M)_j(i)_ is the fixed effect of the dose nested within each metal, and e_ijk_ is the random effect of the residual.

The effects of the organ, metal, and metal doses on the FW, DW, and saponin concentration were evaluated by the lineal model (associated with a mixed two-way ANOVA with nesting):

Y_ijkl_ = µ + O_i_ + M_j_ + D(M)_k(j)_ + OxM_ij_ + e_ijkl_, (3)

where Y_ijkl_ is the measurement in the l-replicate, the i-organ, j-metal, and k-dose nested within each metal. µ is the general mean, O_i_ represents the fixed effect of the organ, M_j_ represents the fixed effect of metal, D(M)_k(j)_ is the fixed effect of the dose nested within each metal, OxM_ij_ is the interaction between both factors, and e_ijkl_ is the random effect of the residual. This model was also utilized to assess the effect of the same factors on the ΔΔCt values in the qPCR analyses.

The statistical analyses were performed using JMP-Genomics 10.1 (SAS, Cary, NC, USA). These included ANOVAs, multiple comparison tests (Least-squares means differences Tukey HSD), and Pearson’s correlations among the traits.

## 5. Conclusions

The Quillay plants exposed to Cd and Cu presented a general defensive mechanism by restricting metal accumulation to the roots, thereby limiting their presence in aerial organs. Metal excess also increased the saponin concentrations, suggesting their role as a defensive mechanism, assuming a metal-binding ability of these metabolites. Gene expression analyses indicated the participation of the antioxidant system (based on glutathione according to our analyses), minimal participation of metal chelating molecules (MTs and PCs), and differential regulation of membrane transporters. These findings highlight specific defensive responses of Quillay to heavy metal stress, providing valuable insights for future research and supporting its potential application in phytoremediation strategies.

## Figures and Tables

**Table 1:** Effect of experimental factors on growth and metal and saponin concentration in Quillay plants. *p*-values from ANOVA are shown. Numbers in bold are significant at α = 0.05.

		*p*-Value		
Trait/Factor	Metal	Dose (Metal)	Organ	Organ × Metal
Height	0.7425	0.8792	-	-
LPR	**0.0179**	**0.0294**	-	-
L/R ratio	**<0.0001**	**0.0004**	-	-
Fresh weight	0.5614	0.4356	**0.0024**	0.5801
Dry weight	0.3408	0.5373	**<0.0001**	0.2098
Cu Conc. ^†^	-	**0.0006**	**<0.0001**	**0.0006**
Cd Conc. ^†^	-	**0.0001**	**<0.0001**	**0.0001**
Saponin Conc.	0.7615	**0.0114**	**<0.0001**	0.6878

Obs.: ^†^ For Cu and Cd concentrations, the ANOVA linear model used “Dose” instead of “Dose(metal)” and “Organ × Dose” instead of “Organ × Metal” as factors.

**Table 2:** Growth and weight traits of Quillay plants under metal stress in terms of treatments and organs. Numbers indicate average and standard deviation (*n* = 3).

		Treatments		Organs	
Trait	Control	Cd ^⁑^	Cu ^⁑^	Leaves	Roots
Height (cm)	33.0 ± 2.3 a	30.9 ± 2.3 a	32.0 ± 2.3 a	-	-
LPR (cm)	17.8 ± 0.8 a	18.8 ± 0.7 b	16.4 ± 0.7 a	-	-
L/R ratio	2.8 ± 0.2 a	1.6 ± 0.1 b	2.8 ± 0.1 a	-	-
Fresh weight (g)	3.5 ± 0.4 a	3.3 ± 0.3 a	3.0 ± 0.3 a	3.7 ± 0.3 A	2.6 ± 0.3 B
Dry weight (g)	1.0 ± 0.2 a	1.1 ± 0.1 a	1.0 ± 0.1 a	1.4 ± 0.1 A	0.7 ± 0.1 B

Obs.: LPR: Length of principal root; L/R: Leaf to root ratio. ^⁑^: Average across the three applied doses for Cd (20, 40, and 80 μM) or Cu (75, 150, and 300 μM). For each trait, different letters indicate significant differences (Least-squares means differences Tukey HSD, *p* < 0.05) among metal doses or organs.

**Table 3:** Metal and saponin concentrations in Quillay plants under metal stress in terms of treatments and organs. Numbers indicate average ± standard deviation (*n* = 3).

			Treatments		Organs	
	Control		Cd	Cu		
Trait	Leaves	Roots			Leaves	Roots
Cu conc. (mg/kg DW)	0.35 ± 0.03 a	3.02 ± 0.66 b	-	217.11 ± 2.16 †	0.43 ± 0.08 A ⁑	433.79 ± 26.95 B ⁑
Cd conc. (mg/kg DW)	0.0 ± 0.0 a	0.0 ± 0.0 a	169.93 ± 2.67 †	-	0.0 ± 0.12 A ⁑	339.90 ± 22.33 B ⁑
Saponin conc. (mM)	11.0 ± 2.4 †		19.3 ± 4.1 † a	20.3 ± 4.1 † b	27.6 ± 1.8 A ⁑	7.6 ± 1.8 B ⁑

Obs.: † Average concentration for leaves and roots; ⁑ Average across the three applied doses for Cu or Cd. For each trait, different letters indicate significant differences (Least-squares means differences Tukey HSD, *p* < 0.05) among metal doses or organs.

**Table 4:** Pearson’s correlation among the assessed traits in Quillay plants exposed to Cu and Cd. Numbers in bold are significant at *α* = 0.05.

	Height	LPR	Lf-FW	Lf-DW	Rt-FW	Rt-DW	L/R Ratio	Lf-sapn	Rt-sapn	Lf-Cu	Rt-Cu	Rt-Cd
Height	-	−0.19	**0.86**	**0.92**	**0.77**	**0.50**	**0.44**	−0.11	0.07	−0.35	−0.11	−0.24
LPR		-	−0.14	−0.24	0.02	0.08	−0.33	0.06	−0.27	−0.14	−0.41	0.19
Lf-FW			-	**0.92**	**0.72**	**0.38**	**0.45**	−0.32	0.32	−0.44	−0.35	−0.40
Lf-DW				-	**0.80**	**0.48**	**0.42**	−0.05	0.23	−0.41	−0.12	−0.16
Rt-FW					-	**0.73**	0.03	0.03	0.08	−0.25	−0.10	0.25
Rt-DW						-	**−0.55**	0.09	−0.10	−0.33	−0.14	**0.47**
L/R ratio							-	−0.26	0.19	−0.09	−0.01	**−0.70**
Lf-sapn								-	**−0.42**	−0.07	**0.56**	**0.62**
Rt-sapn									-	−0.27	**−0.47**	−0.34
Lf-Cu										-	**0.59**	0.00
Rt-Cu											-	0.00
Rt-Cd												-

Obs.: LPR, length of principal root; Lf-FW, leaf fresh weight; Lf-DW, leaf dry weight; Rt-FW, root fresh weight; Rt-DW, root dry weight; L/R ratio, leaf to root ratio; Lf-sapn, leaf saponins; Rt-sapn, roots saponins; Lf-Cu, Cu in leaves; Rt-Cu, Cu in roots; Rt-Cd, Cd in roots.

**Table 5:** Effect of experimental factors on gene expression in Quillay plants. *p*-values from ANOVA are shown. Numbers in bold are significant at *α* = 0.05. *GS*, glutathione synthetase; *GR*, glutathione reductase; *COPT1*, copper transporter; *PCS*, phytochelatin synthase; *MT*, metallothionein.

			*p*-Value	
Gene/Factor	Organ	Metal	Dose (Metal)	Organ × Metal
*GR*	**0.01392**	**0.04059**	0.07509	0.55948
*GS*	**0.00248**	0.17322	0.23309	0.46768
*COPT1*	**0.00244**	0.9332	**0.00614**	0.42833
*PCS*	0.24323	0.78324	0.20516	0.54845
*MT*	N.E.	**<0.0001** †	0.3858	N.E.

Obs.: N.E., non-estimated; † Estimated only for leaves.

## Data Availability

Data supporting the reported results that are not included in the Supplementary Materials can be requested from the authors.

## Supplementary Materials

The following supporting information can be downloaded at: https://www.mdpi.com/article/10.3390/plants14050709/s1, Table S1: Concentration of Cu and Cd in certified reference material; Table S2: Primer sequences, melting temperature, and amplicon length; Figure S1: Transcriptional variation of *COPT1* gene in roots of quillay plants exposed to Cd and Cu; Figure S2: Transcriptional variation of *MT* gene in quillay plants exposed to Cd and Cu; Figure S3: Pearson’s correlation among the assessed gene transcripts in quillay plants exposed to Cu and Cd; Figure S4: Transcriptional variation of *IRT1* gene in roots of quillay plants exposed to Cd and Cu.
