# Supplementary material for: Cadmium and Copper Stress Responses in Soapbark Tree (Quillaja saponaria): Effects on Growth, Metal Accumulation, Saponin Concentration, and Gene Expression
Source: Plants (Basel). 2025 Feb 26;14(5):709. doi: 10.3390/plants14050709 (PMC11901668; doi:10.3390/plants14050709)
Supplement: Supplementary file 1 [file plants-14-00709-s001.zip › plants-3460191-supplementary.pdf]

Supplementary Materials

Table S1. Concentration of Cu and Cd (mg/kg) in certified reference material (BIMEP-432) from the Wageningen Evaluating Programs for Analytical Laboratories (WEPAL).

Element	Certified Concentration and MAD[a]	Observed Concentration (n=3) and std. deviation	Rel. Error (%)	Recovery (%)
Cu	6.05 (3.50) (n= 6)	6.17 ± 0.04	1.98	+ 101.98
Cd	1.30 (0.90) (n= 4)	1.26 ± 0.01	3.08	- 96.92

Obs.: a) In parenthesis, MAD indicates median absolute deviation

Table S2. Primer sequences, melting temperature (tm), and amplicon length.

Gen	Primer		tm (°C)	Amplicon (bp)
Metallothionein (*MT*)	Fw	TGTGAGTGCAACCCATGCAG	58.1	78
	Rv	CACAAGTGCAAGATGCGTCAC	57.9	
Phytochelatin synthase (*PCS*)	Fw	GAGCTGCAAACACGAGAGTTG	57.3	86
	Rv	CCTGATCTCTGCAACCCATTTG	57.0	
Glutathione synthase (*GS*)	Fw	CGATGTGAGAAAAGCCCTCCT	57.27	85
	Rv	GCCAACAGCCATTACGCATC	57.29	
Glutathione reductase (*GR*)	Fw	AAGGCGAGGGAAAGATAGTTGG	57.52	83
	Rv	TGTGCTTTGCTGTGTAGGACA	57.34	
Copper transporter (*COPT1*)	Fw	CGTCATGTCGGTTTTCGTGC	57.56	114
	Rv	GAGACCAACCCTGAAAGCGA	56.93	

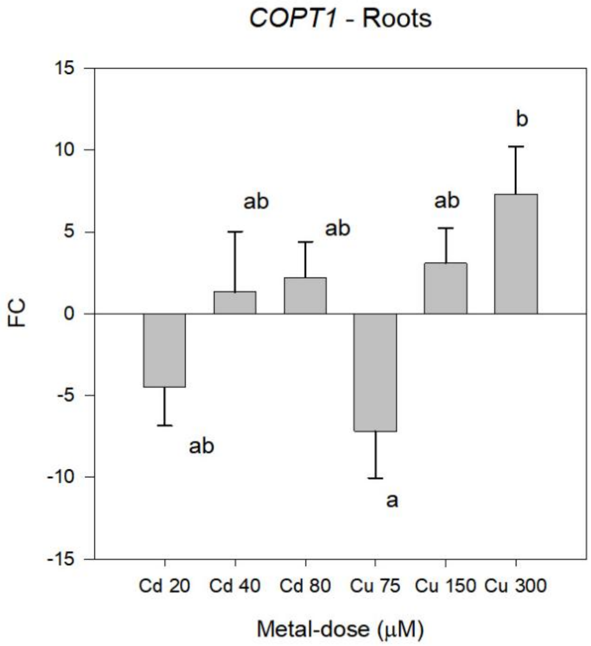

Figure S1. Transcriptional variation of the *COPT1* gene in Quillay root tissues exposed to Cd and Cu. FC represents the fold change relative to control plants. Different letters indicate significant differences among metal doses (LSMeans Differences Tukey HSD, p-value < 0.05). In contrast, leaf gene expression showed no significant differences (p-value = 0.5978) across the evaluated metal doses.

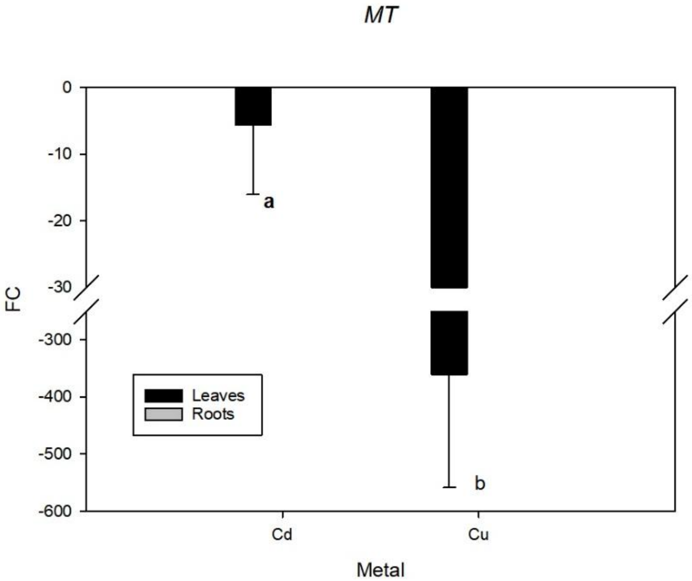

Figure S2. Transcriptional variation of *MT* gene in quillay plants exposed to Cd and Cu. This assessment was only possible for leaves since no transcripts of *MT* were detected in roots. FC represents the fold of change concerning control plants. Different letters indicate significant differences (LSMeans Differences Tukey HSD, *p-value* < 0.05) among metals.

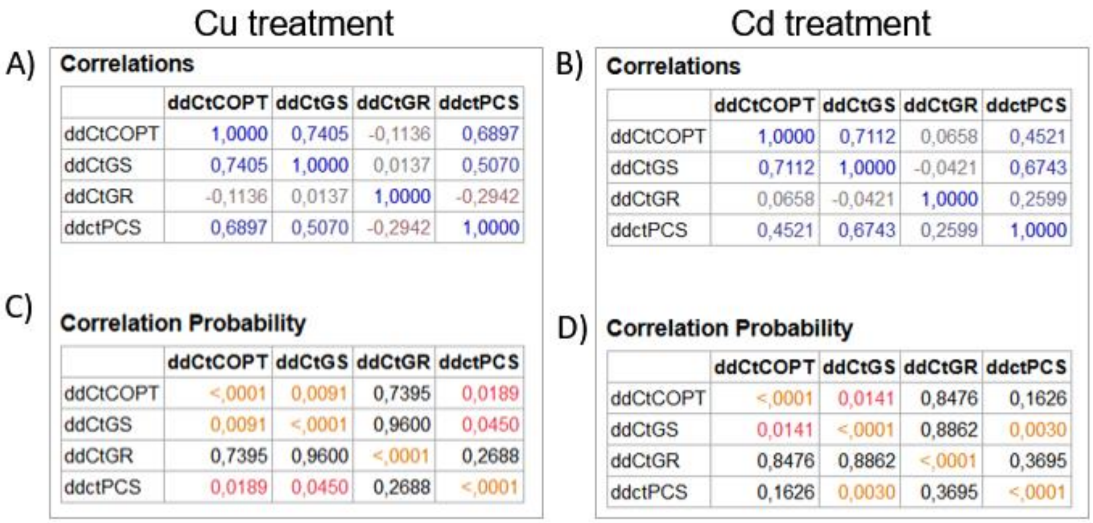

Cu treatment

A) Correlations

	ddCtCOPT	ddCtGS	ddCtGR	ddctPCS
ddCtCOPT	1,0000	0,7405	-0,1136	0,6897
ddCtGS	0,7405	1,0000	0,0137	0,5070
ddCtGR	-0,1136	0,0137	1,0000	-0,2942
ddctPCS	0,6897	0,5070	-0,2942	1,0000

C) Correlation Probability

	ddCtCOPT	ddCtGS	ddCtGR	ddctPCS
ddCtCOPT	<,0001	0,0091	0,7395	0,0189
ddCtGS	0,0091	<,0001	0,9600	0,0450
ddCtGR	0,7395	0,9600	<,0001	0,2688
ddctPCS	0,0189	0,0450	0,2688	<,0001

Cd treatment

B) Correlations

	ddCtCOPT	ddCtGS	ddCtGR	ddctPCS
ddCtCOPT	1,0000	0,7112	0,0658	0,4521
ddCtGS	0,7112	1,0000	-0,0421	0,6743
ddCtGR	0,0658	-0,0421	1,0000	0,2599
ddctPCS	0,4521	0,6743	0,2599	1,0000

D) Correlation Probability

	ddCtCOPT	ddCtGS	ddCtGR	ddctPCS
ddCtCOPT	<,0001	0,0141	0,8476	0,1626
ddCtGS	0,0141	<,0001	0,8862	0,0030
ddCtGR	0,8476	0,8862	<,0001	0,3695
ddctPCS	0,1626	0,0030	0,3695	<,0001

Figure S3. Pearson's correlations among the assessed gene transcripts in soapbark plants exposed to Cu and Cd. A) and B) Correlation coefficients for plants exposed to Cd and Cu, respectively. C) and D) P-values for correlation coefficients for plants exposed to Cu and Cd, respectively.

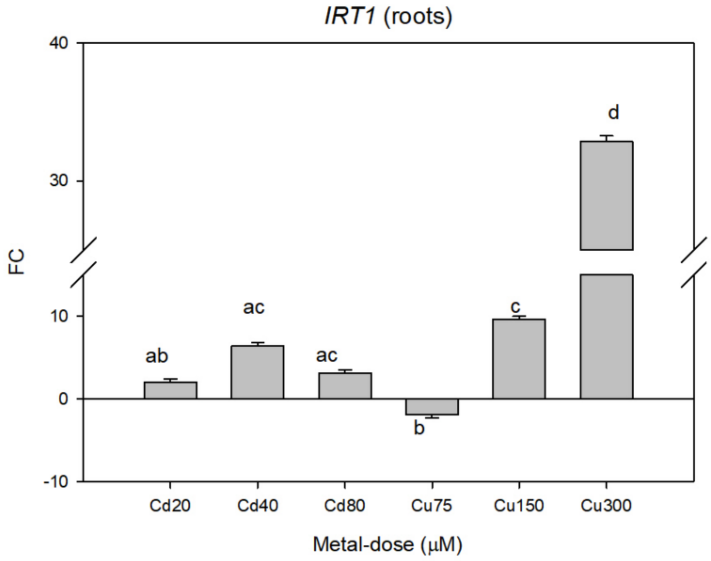

Figure S4. Transcriptional variation of *IRT1* in roots of quillay plants exposed to Cd and Cu. FC represents the fold of change concerning control plants. Different letters indicate significant differences (LSMeans Differences Tukey HSD, *p-value* < 0.05) among metal doses.
